# Machine Learning-Driven Prediction of Brain Age for Alzheimer’s Risk: APOE4 Genotype and Gender Effects

**DOI:** 10.3390/bioengineering11090943

**Published:** 2024-09-20

**Authors:** Carter Woods, Xin Xing, Subash Khanal, Ai-Ling Lin

**Affiliations:** 1 Department of Physics, University of Missouri, Columbia, MO 65211, USA; 2 Department of Computer Science, University of Nebraska Omaha, Omaha, NE 68182, USA; xxing@unomaha.edu; 3 Department of Computer Science & Engineering, Washington University in St. Louis, St. Louis, MO 63130, USA; 4 Department of Radiology, Biology and Institute for Data Science and Informatics, University of Missouri, Columbia, MO 65211, USA; 5 Sanders-Brown Center on Aging, Department for Pharmacology and Nutritional Sciences, University of Kentucky, Lexington, KY 40506, USA

**Keywords:** brain age prediction, Alzheimer’s disease, apolipoprotein E4 alleles, magnetic resonance imaging, machine learning, random forest, XGBoost, regression models, cross-validation

## Abstract

**Background:** Alzheimer’s disease (AD) is a leading cause of dementia, and it is significantly influenced by the apolipoprotein E4 (APOE4) gene and gender. This study aimed to use machine learning (ML) algorithms to predict brain age and assess AD risk by considering the effects of the APOE4 genotype and gender. **Methods:** We collected brain volumetric MRI data and medical records from 1100 cognitively unimpaired individuals and 602 patients with AD. We applied three ML regression models—XGBoost, random forest (RF), and linear regression (LR)—to predict brain age. Additionally, we introduced two novel metrics, brain age difference (BAD) and integrated difference (ID), to evaluate the models’ performances and analyze the influences of the APOE4 genotype and gender on brain aging. **Results:** Patients with AD displayed significantly older brain ages compared to their chronological ages, with BADs ranging from 6.5 to 10 years. The RF model outperformed both XGBoost and LR in terms of accuracy, delivering higher ID values and more precise predictions. Comparing the APOE4 carriers with noncarriers, the models showed enhanced ID values and consistent brain age predictions, improving the overall performance. Gender-specific analyses indicated slight enhancements, with the models performing equally well for both genders. **Conclusions:** This study demonstrates that robust ML models for brain age prediction can play a crucial role in the early detection of AD risk through MRI brain structural imaging. The significant impact of the APOE4 genotype on brain aging and AD risk is also emphasized. These findings highlight the potential of ML models in assessing AD risk and suggest that utilizing AI for AD identification could enable earlier preventative interventions.

## 1. Introduction

Alzheimer’s disease (AD) is the most prevalent form of dementia, affecting an estimated 32 million people worldwide. Additionally, about 384 million individuals are on the broader spectrum of AD, impacting over one-fifth of the population aged 50 and older [1]. With an aging population, the number of affected individuals is expected to rise, and the economic burden is already significant—AD and other dementias currently cost the United States alone USD 196 billion annually [2].

Characterized by progressive cognitive decline and memory loss, AD is marked by an accelerated reduction in brain volume, especially in the hippocampus, a region crucial for memory and cognition [3,4,5,6,7,8]. Key indicators include the presence of the apolipoprotein E4 (APOE4) gene, which is the primary genetic risk factor for AD, and gender disparities, with women being at a higher risk than men [9,10]. These factors contribute to more pronounced atrophy in specific brain regions for APOE4 carriers and manifest differently between the sexes [3,4,10,11,12].

While the absence of a cure has highlighted the failures of post-symptomatic treatments, it has also spurred a significant expansion in research focused on presymptomatic treatment and diagnosis [13]. A key factor in this approach is the early identification of accelerated brain aging, which is crucial for enabling timely interventions that could slow the progression of Alzheimer’s disease (AD). Magnetic resonance imaging (MRI) plays a pivotal role by revealing subtle structural changes in the brain, thereby facilitating the early detection of AD risk and other neurodegenerative diseases [11,14,15].

Numerous studies have utilized the abundant longitudinal data from electronic health records (EHRs), either independently or in conjunction with MRI data, to train AI models aimed at the early prediction of Alzheimer’s disease [16,17,18,19,20,21,22]. These models have demonstrated impressive results in correlating AD with specific brain structure volumes and other medical information, with some achieving diagnostic accuracy that surpasses that of neuroradiologists. However, a significant challenge with these classification models is their tendency to provide binary or graded diagnoses, which may not adequately capture the gradual and heterogeneous progression of the disease among patients.

The potential of using MRI to develop models that predict “brain age”—a continuous measure that could serve as an indicator of increased AD risk—remains largely underexplored. This study aims to fill this knowledge gap by investigating the effectiveness of patient-specific training and by implementing novel statistical tools for model selection and assessment.

The study had the following two primary objectives: first, to develop machine learning (ML) algorithms capable of determining brain age relative to chronological age and, second, to identify effective models and training procedures that can stratify APOE4 genotype and gender effects in assessing AD risk. Our hypotheses are that (i) accelerated brain aging can accurately indicate a higher risk of AD; (ii) MRI brain volume data can be used to estimate brain age; (iii) APOE4- and gender-specific training will improve predictions of brain age; and (iv) APOE4 carriers and females may experience more rapid brain volume reductions compared to APOE4 noncarriers and males.

Machine learning (ML)-driven algorithms have been widely employed for predicting AD risk [23,24]. However, using ML to predict brain age is a relatively novel approach. A recent study showed that ML-based linear regression (LR) can effectively predict brain age for epilepsy [25]. In our research, we expanded on this by incorporating the following two additional regression models: XGBoost and random forest (RF). We compared their efficacies with LR in determining brain age from MRI volumetric data, leading to the development of mathematical models that describe the predictive capabilities of these algorithms. We introduced the following two new metrics: “brain age difference” (BAD) and ”integrated difference” (ID), which enhanced the comparison of brain ages between healthy individuals and patients with AD, offering insights into the progression of brain atrophy. These methods were also applied to explore the influence of the APOE4 variant and gender on brain atrophy. Our goal is to identify the most precise and efficient ML model and training methods for AD detection, with significant implications for future clinical applications.

## 2. Materials and Methods

### 2.1. Data

We obtained brain volumetric MRI data and medical information from the National Alzheimer’s Coordinating Center (NACC), a publicly available database [26]. The dataset comprised a total of 1702 participants, including 1,100 cognitively unimpaired (CU) individuals and 602 with Alzheimer’s disease (AD). Table 1 presents the subjects’ demographics, with the *p*-values computed against the null hypothesis that these characteristics are uncorrelated with an Alzheimer’s diagnosis. Both study groups were matched for age (CU: 76.1 ± 8.3 years; AD: 76.1 ± 8.5 years; *p* = 0.93). However, differences were observed in educational attainment, with the CU group having a higher average level of education. Additionally, a greater proportion of females was noted in the CU group. Importantly, significant differences were found among the groups in the presence of the ε4 allele of apolipoprotein E (APOE ε4), a major genetic risk factor for Alzheimer’s [9,23,27,28]. Participants in the AD group were notably more likely to carry the APOE ε4 allele compared to those in the CU group.

Each participant’s dataset consisted of 175 features, including 19 medical attributes and 156 brain volumetric measurements. A comprehensive list of these features is available in Table S1. The brain volumetric data revealed significant differences in several key brain regions associated with cognitive function, with patients with AD exhibiting significantly lower values compared to CU participants. Table 2 ranks these features by their statistical significance, computed against the null hypothesis that these features are uncorrelated with an Alzheimer’s diagnosis. We utilized this data for machine learning (ML) training to determine brain age differences that could predict AD risk in aging individuals.

### 2.2. Architecture and Training Procedure

**Figure 1:**
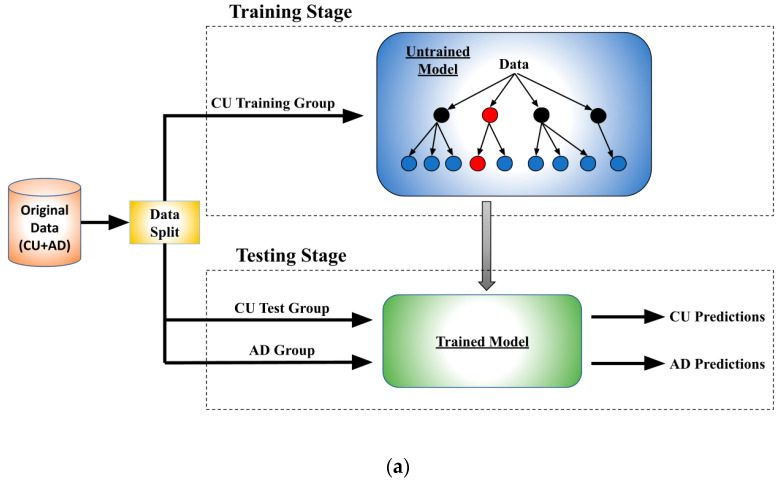

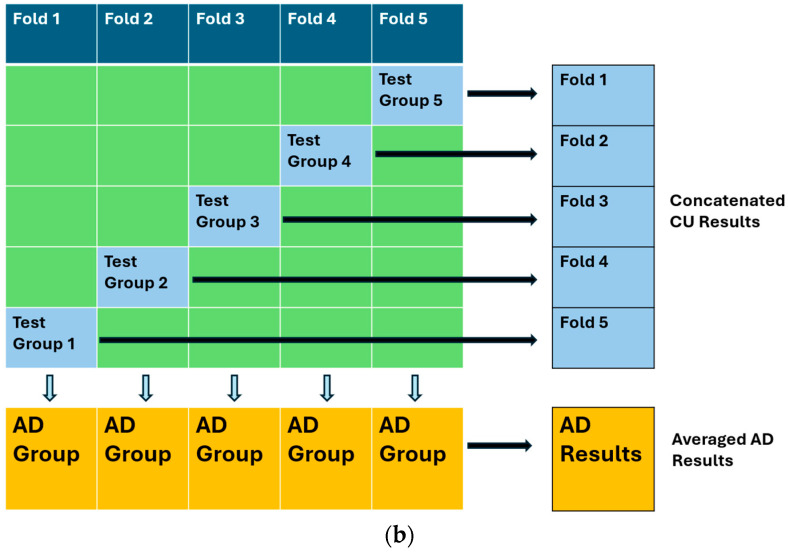
(**a**) Workflow and data splitting within each fold; (**b**) 5-fold validation method used within each iteration.

We used the following three distinct ML regression models in brain age prediction for comparative purposes: extreme gradient boosting (XGBoost), random forest (RF), and a simple linear regression (LR). For optimizing model parameters, we implemented a cross-validated grid search algorithm specifically for XGBoost and RF. Figure 1a illustrates the overall architecture and training workflow.

To establish a baseline, each model was initially trained exclusively on CU data. We implemented 5-fold cross-validation to mitigate randomness and ensure comprehensive testing across the entire dataset. Before training, the CU data was divided into a training subset (80%) and a testing subset (20%). In each of the five folds, a different 20% of the CU data was excluded from the training set and used as the testing set. The process of training and testing within a single fold is illustrated in Figure 1a.

During the training stage, models were trained on the designated training subset. Upon completing their training, each model was then applied to both the patients with AD dataset and the CU testing subset to predict brain age. In the subsequent 5-fold iterations, depicted in Figure 1b, 20% of the CU data was removed for testing in each iteration, allowing for thorough testing across the entire available CU data and repeated testing over the AD group. The results from these iterations were averaged after each fold. In total, 25 iterations were performed, with the results averaged again to further reduce randomness.

For trials focusing on the APOE ε4 allele and gender differences, we employed three distinct training procedures, as detailed in Section 3.3.1 and Section 3.4.1. Each procedure stratified the CU training group based on either gender or genotype.

We introduced a novel metric, brain age difference (BAD), to assess the degree to which a model estimates individuals with AD to be older than CU individuals. To calculate a model’s BAD, we first established the lines of best fit (LOBFs) that characterize the prediction distribution for the model-predicted ages for the CU and AD groups. We then integrated the area between these LOBFs across the target population’s age range and divided this integral by the age range to derive the BAD, as depicted in Equation (1) where the variable x represents an individual’s real chronological age.

(1) $$BAD=\frac{\int_{55}^{75}(AD\;PREDICTION\;LOBF\left(x\right))-(CU\;PREDICTION\;LOBF\left(x\right))dx}{\left(75-55\right)}$$

Describing the separation between the CU and AD prediction distributions, a large BAD is attributed to an effective diagnostic model. For a single individual, their BAD would be the difference between their model-predicted age and the normal-model-predicted age for a CU individual the same age, with higher BADs indicating higher AD risk. The uneven age distribution within the target region led us to calculate a model’s BAD using Equation (1) instead of simply averaging the individuals’ BADs so that the higher concentration of individuals at the upper age limit of the target region would not dominate the calculation of a model’s BADs. A model with a higher BAD indicates a larger AD risk when tested on patients diagnosed with AD.

Despite its utility, BAD is only a baseline value and does not gauge a model’s precision. To complement this, we introduced the integrated difference (ID) values, a novel statistical method that provides a measure of precision, as outlined in Equation (2). Similarly to Equation (1), x represents real chronological age, while y represent an individual’s model predicted.

(2) $$ID=\frac{\int_{55}^{75}\int_{-\infty }^{\infty }\left|\left(AD\;PDF(x,y\right))-\left(CU\;PDF(x,y\right))\right|dy\;dx}{40}$$

**Figure 2:**
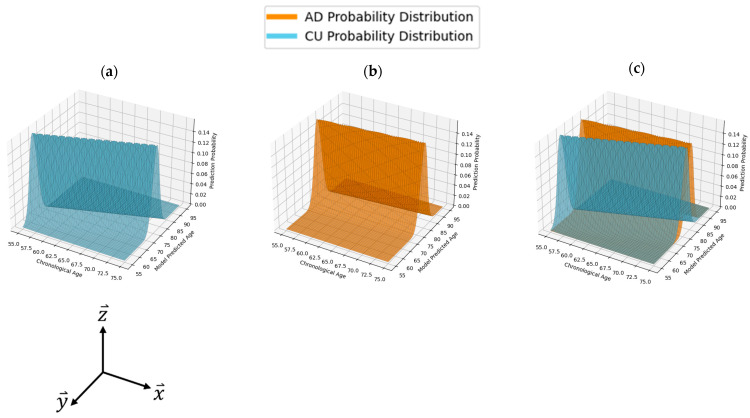
Probability distribution functions (PDFs) modeled to ref. CU and AD prediction distributions: (**a**) PDF of age predictions for CU individuals; (**b**) PDF of age predictions for patients with AD; (**c**) two PDFs overlaid on each other. The z-axis describes the probability of a model making a prediction for a given individual’s chronological age. Similarly to further figures, the x-axis represents an individual’s chronological or real age, while the y-axis describes their model-predicted age.

The use of ID values in this study served to more accurately capture the probabilistic nature of the regression models while assessing their effectiveness in distinguishing between the following two populations: CU and AD. The ID values achieve this by generating PDFs for each model’s age prediction distribution. These PDFs, visualized in Figure 2, allow for the computation of the probability of an individual’s AD diagnosis based on their projected brain age.

To generate these PDFs, we first utilized the standard deviation (STD) of each model’s prediction distribution about its LOBF. The STD is then used to construct normal PDFs describing the behavior of the CU and AD predictions, as displayed in Figure 2. We then calculated the absolute difference among these PDFs and normalized the result, yielding a value that reflects the degree of overlap between the two distributions. The ID value ranges from 0 to 1, where a value of 0 indicates two identical prediction PDFs, and a value of 1 represents completely distinct distributions with no overlap. A model with an ID value of 1 would theoretically predict an individual’s mental health status with perfect accuracy. Conversely, an ID value of 0 would be found for a model with no ability to distinguish between CU and AD individuals. ID comprehensively describes the clinical applicability of a model by directly describing the difference between CU and AD functions and was used as the primary metric for model evaluation throughout this study.

## 3. Results

### 3.1. Model Performances on All Subjects 

**Figure 3:**
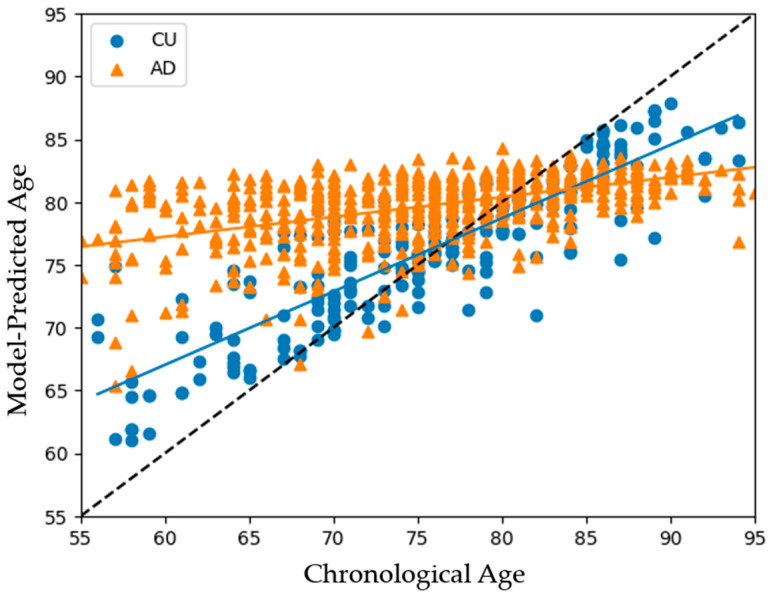
Model results when tested on the full age range of the subjects with AD. Individuals’ real chronological ages are on the x-axis and their model-predicted age on the y-axis. The crossing of the distribution LOBFs demonstrates the model’s deprecating ability to make meaningful predictions for AD subjects at higher ages.

Figure 3 presents the brain estimate age versus chronological age results for all subjects, including the 1100 CU individuals and 602 patients with AD. The black dashed line represents the regression line between each individual’s chronological age and their estimated brain age. Data points above this line indicate an older brain age relative to chronological age, and points below indicate a younger brain age. The LOBF for CU and AD are shown in blue and orange, respectively. The graph illustrates that patients with AD typically exhibit significantly older brain ages compared to CU individuals between 55 and 75 years of age, with BADs of 6.5–10 years separating the two groups. These highlight the accelerated brain aging in patients with AD relative to their chronological age.

Our analysis reveals that the models progressively lose their ability to effectively differentiate between individuals with AD and CU with age. The BADs steadily decreased with age before becoming negative, as the models began to predict lower ages for individuals with AD than for CU at 80–85 years old. This suggests that the models become less capable of discerning differences from MRI brain imaging at higher ages, regardless of the individual’s health status. Given that our goal is to develop tools for the early detection of AD, this limitation is not critical for evaluating individuals within the primary target group of ages 55 to 75. To more precisely assess the models’ performances in this targeted age range, we conducted tests using the various models on individuals within this demographic. The training continued with the 1100 CU individuals but within the testing range of 55–75 years of age; 451 CU individuals and 238 patients with AD were evaluated.

### 3.2. Comparisons among the Three ML Models

**Figure 4:**
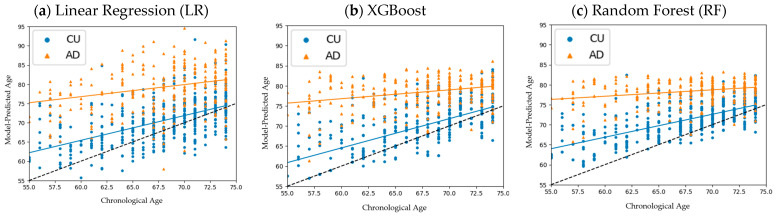
Age prediction distributions: (**a**) linear regression; (**b**) XGBoost; (**c**) random forest. RF showed tighter predictive distributions around its LOBFs compared to LR and XGBoost.

We first compared the following three models: RF, XGBoost, and LR. The BAD, ID value, and STD of the CU and AD prediction distributions are shown in Table 3, with the STDs being calculated about each distribution’s respective LOBF. We found that the ID values of RF (0.762) and XGBoost (0.75) were far larger than that of linear regression (0.604), indicating clearer separation between the CU and AD distributions using RF and XGBoost. The corresponding scatterplots are shown in Figure 4; LR had the largest variations in age predictions, while RF returned the lowest STDs for its predictions. Despite the RF model generating smaller BADs, its tighter distributions led it to generate the highest ID values among the three models.

**Figure 5:**
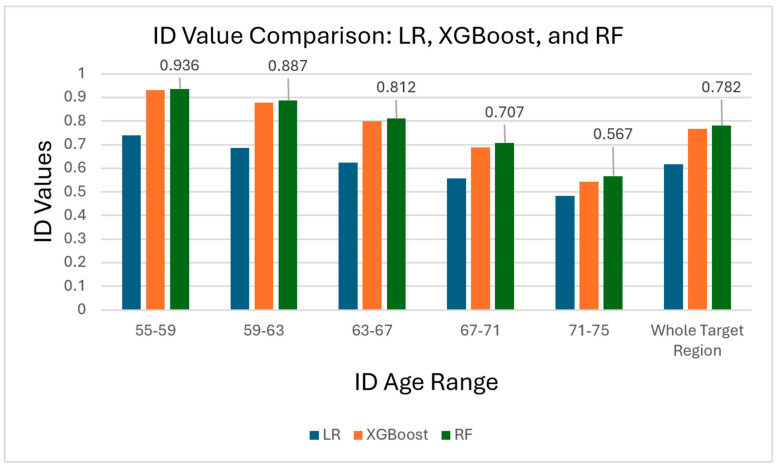
Histogram comparing ID values of each of the three models over each calculated age range. Shown is the highest ID calculated across each region, generated by RF in each case.

The ID values were further examined in smaller four-year increments (Table 4). Figure 5 compares the ID values calculated for each model across the different age ranges. These incremental ID values indicate that XGBoost performed marginally better at the lower end of the target age range, while RF scored significantly higher in ID values for age groups, where the concentration of tested individuals was greatest. Given its highest ID values and superior precision, we concluded that RF was the best model for indicating AD and, therefore, continued using RF for the remainder of the study.

### 3.3. APOE4 Comparison

#### 3.3.1. APOE4-Stratified Model Compositions

We stratified the dataset based on APOE4 status and applied five different training models to test the effect of APOE4- and genotype-specific training on the model performance (Table 5). The data was first split, as described in Section 2.2, before being further split as described below:

**Model A (E4-Specific):** trained with and tested on 100% E4-carriers (N = 351);

**Model B (E4-Specific):** trained with and tested on 100% E4 noncarriers (N = 749);

**Model C (Mixed):** trained with a mixed dataset of E4-carriers and NCs (E4-carrier: N = 351; E4-NC: N = 749) and tested over both the E4-carrier and E4-NC groups;

**Model D (Mixed-Condensed):** Trained with an evenly mixed dataset of E4-carriers and NCs (E4-carrier: N = 176; E4-NC: N = 176) matched in sized to Model A to examine the effects of APOE4-specific training, specifically on E4-carriers. Tested on both E4-carriers and E4-NCs to examine the model effectiveness on both groups given the equal training size;

**Model E (Mixed-Condensed):** Trained with an evenly mixed dataset of E4-carriers and NCs (E4-carrier: N = 351; E4-NC: N = 398), matched in sized to Model B to directly examine the effects of APOE4-specific training, specifically on E4-NCs. Tested on both E4-carriers and E4-NCs to examine the model effectiveness on both groups given the similar training sizes.

The desired 50–50 ratio of E4-carriers to E4-NCs was not possible for Model E. Model E was created to match Model B’s number of individuals in the training set (N = 749); however, there were too few E4-carriers (N = 351) to compose half of the training group.

#### 3.3.2. APOE4-Stratified Model Outcomes

Table 6 shows the testing outcomes. We found that the APOE4 testing resulted in the highest model performance for all training methods. Among E4-specific models A and B, model A generated a higher ID score despite being trained on a smaller set of data. In mixed-training model C, we similarly see a higher performance on E4-carriers despite being trained on a higher number of E4-NCs than E4-carriers.

The higher performances on APOE4 carriers were most evident among mixed-condensed models D and E, which were both trained on even or near even numbers of E4-carriers and E4-NCs. These models generated ID values that were %35 (model D) and %17 higher (model E) when tested on E4-carriers compared to NCs, showing significant increases in model differentiation between the CU and AD groups.

Comparing the models trained on equally sized datasets, model A displayed no performance benefits over model D when tested on E4-carriers. Oppositely, model B generated an ID value %7 higher than its mixed-condensed counterpart model E when tested on E4-NCs. These results suggest that patient genotype-specific training may only be advantageous for E4-NCs. They further suggest a more homogenous progression of brain atrophy among CU E4-NCs compared to their E4-carrier counterparts.

Predictably, the models demonstrated better performances when tested on larger datasets. Observing the models tested on both E4-carriers and E4-NCs, the combined ID values generated for both groups were the largest for model C, second largest for model E, and third largest for model D, following the overall training sizes for each group. These results reinforce the importance of greater availability of data for model performance.

### 3.4. Gender Comparison

#### 3.4.1. Gender-Stratified Model Compositions

To similarly compare the effects of gender on model performance, we stratified our training set into five different groups (A–E) by gender; the composition of each group is displayed in Table 7.

**Model A (Female-Specific)** trained and tested on 100% females (N = 705);

**Model B (Male-Specific):** trained and tested on 100% males (N = 395);

**Model C (Mixed):** trained on a mixed dataset of males and females (Female: N = 705; Male: N = 395) and tested separately on both males and females;

**Model D (Mixed-Condensed):** trained on an evenly mixed dataset of females and males (Female: N = 353; Male: N = 353), matched in total size to model A to examine the effects of gender-specific training, specifically on females. Tested on both females and males to examine the model effectiveness on both groups given the similar training sizes;

**Model E (Mixed-Condensed):** trained on an evenly mixed dataset of females and males (Female: N = 198; Male: N = 198), matched in total size to model B to directly examine the effects of gender-specific training, specifically on males. Tested on both females and males to examine the model effectiveness on both groups given the similar training sizes.

#### 3.4.2. Gender-Stratified Model Outcomes

The training outcomes are summarized in Table 8. Model C consistently produced the highest ID values, demonstrating that models with larger training groups tend to perform better. Individualized training also proved to be advantageous, with gender-specific models A and B outperforming the similarly sized mixed-condensed models D and E on their respective test groups. Notably, these results highlight that E4-carriers were the only group that did not benefit from specified training, suggesting underlying non-homogeneity in brain atrophy among CU E4-carriers. Additionally, the overall lower impact of gender stratification on model performance indicates that gender is a weaker risk factor in this context.

Unlike in the APOE4-stratified trial, neither males nor females led to significantly higher performances when tested upon. The gender-specific models (A and B) and the general mixed model (C) all generated higher ID values for females; however, these models either had larger training sizes of females or a higher percentage of females in the training group. The evenly gender-stratified mixed-condensed models (D and E) opposed each other, with model D performing better on females and model E on males.

## 4. Discussion

In this study, we used ML algorithms to predict brain age and assess AD risk, and there are four key findings. First, patients with AD exhibited significantly older brain ages compared to CU individuals, with BADs, ranging from 5 to 9.7 years, indicating accelerated brain aging. Second, we compared the performance of the following three ML algorithms: LR, XGBoost, and RF. Unlike previous studies that used LR to predict brain age [25], our results reveal that RF consistently outperformed both XGBoost and LR by generating tighter predictive distributions and higher integrated difference (ID) values. Third, models trained with APOE4 data showed enhanced performance on APOE4 carriers compared to noncarriers, with higher ID values and more consistent brain age predictions. Training over the entire dataset (model C) yielded the best overall results, while patient-specific training returned higher ID values for all groups besides the APOE4 carrier group. The advantages of patient-specific training are expected to increase with greater data availability, while the drawbacks of reduced training dataset sizes are expected to decrease. Compared to significant advantages for APOE4 carriers, the results indicate little difference between male and female brain age predictions, suggesting that gender is a weaker risk factor and training condition compared to the APOE4 genotype.

A unique aspect of this study is the implementation of novel ID values, which allowed for a more effective evaluation of the models’ predictive capabilities and an individual’s AD risk. The probability density functions (PDFs) constructed to calculate the models’ ID values can be utilized in future research to determine the probability of an AD diagnosis, offering a continuous metric that indicates an individual’s position on the AD spectrum. Additionally, ID values enable the examination of model performance over smaller segments within a target age range, facilitating a more detailed analysis. Future studies could benefit from patient-specific training and the development of more sophisticated PDF derivations. The method used to construct PDFs in this study assumes a static standard deviation (STD) around the line of best fit (LOBF). Adopting a more dynamic approach to PDF calculation could further enhance model evaluations in future research.

The current study has a few limitations. Firstly, there is a gender imbalance in our data, with a larger proportion of females in the CU group and fewer females than males in the AD diagnosed group. This does not align with established trends, which indicate that women are at a higher risk of AD. This discrepancy is likely due to selection bias in the NACC data, which was compiled from various clinical trials. Secondly, our models became less effective at distinguishing brain ages between individuals with AD and those CU over 80 years old. This could be attributed to the increased brain atrophy that naturally occurs in individuals over 80, reducing the models’ effectiveness. Future studies may aim for a balanced gender distribution and develop age-specific models to improve accuracy.

Alzheimer’s disease is characterized by extracellular beta amyloid (Aβ) plaques (A), intraneuronal tau tangles (T), and neurodegeneration (N), referred to as the A/T/N framework for AD biomarker classification [29,30]. Brain volume atrophy, assessed by MRI, is part of the “N” marker. Our current findings align with existing literature suggesting that “N” markers may be more effective in early prediction of Alzheimer’s disease (AD) progression than “A” and “T” markers [31,32]. Early prediction and diagnosis can enable timely interventions for mitigating or preventing AD risk. For instance, animal models have shown that through either pharmacological or nutritional interventions such as mTOR inhibition can protect against brain aging and reduce AD risk, particularly for APOE4 carriers and females [33,34,35,36,37,38,39,40]. By combining early prediction of brain age using machine learning with potential interventions, the risk of AD could be further mitigated and prevented.

In summary, the study’s implications for future clinical settings are significant. MRI-based ML models, particularly RF, can be integrated into clinical practice to identify individuals at higher risk for AD earlier, enabling timely intervention and potentially slowing disease progression. The ability to predict brain age and stratify risk based on the APOE4 genotype and gender can lead to more effective and personalized diagnostic and treatment plans. With increased data availability, individualized training can be utilized to continuously enhance the model performance. Regular brain age assessments using ML models can monitor disease progression and the effectiveness of interventions over time. Overall, the study highlights the potential of MRI-based ML models in improving AD diagnosis and management, advocating for further research to refine these models and better understand the biological factors influencing brain aging.

## Figures and Tables

**Table 1:** Subject sampling results.

Subject Characteristic	CU	AD	*p*-Value
Number	1100	602	
APOE4 (% Carrier)	32%	58%	<0.001
Age	76.1 ± 8.3	76.1 ± 8.5	0.93
Gender (% Female)	64%	47%	<0.001
Education	15.5 ± 3.6	14.7 ± 3.8	<0.001

**Table 2:** Most significant differences volumetric features.

Feature Rank	Feature Description	CU (mean ± STD)	AD (mean ± STD)	*p*-Value
1	Right entorhinal mean cortical thickness (mm)	3.76 ± 0.58	2.80 ± 0.86	<0.001
2	Left entorhinal mean cortical thickness (mm)	3.56 ± 0.62	2.73 ± 0.80	<0.001
3	Segmented total hippocampi volume (cc)	6.28 ± 0.39	5.37 ± 1.00	<0.001
4	Segmented left hippocampus volume (cc)	3.11 ± 0.30	2.63 ± 0.52	<0.001
5	Left isthmus cingulate mean cortical thickness (mm)	2.30 ± 0.30	1.97 ± 0.35	<0.001
6	Segmented right hippocampus volume (cc)	3.19 ± 0.39	2.73 ± 0.53	<0.001
7	Right superior temporal mean cortical thickness (mm)	2.23 ± 0.30	1.90 ± 0.30	<0.001
8	Right isthmus cingulate mean cortical thickness (mm)	2.33 ± 0.31	2.00 ± 0.38	<0.001
9	Right fusiform mean cortical thickness (mm)	2.56 ± 0.48	2.13 ± 0.42	<0.001
10	Left superior temporal mean cortical thickness (mm)	2.12 ± 0.25	1.85 ± 0.33	<0.001

**Table 3:** Differentiation metrics.

Model Type	BAD	CU (STD)	AD (STD)	ID
Linear Regression	9.70	4.9	6.1	**0.618**
XGBoost	9.72	4.0	3.6	**0.768**
Random Forest	8.2	3.2	3.0	**0.782**

**Table 4:** Limited range ID values.

Years of Age	55–59	59–63	63–67	67–71	71–75
Linear Regression ID	0.739	0.686	0.625	0.558	0.484
XGBoost ID	0.930	0.878	0.799	0.689	0.544
Random Forest ID	0.936	0.887	0.812	0.707	0.567

**Table 5:** Training group compositions for the APOE4 comparison.

Training Group	Training Method	Training Size	Training Group Makeup
A	E4-Specific	351	351 E4-carriers, 0 E4-NCs
B	E4-Specific	749	0 E4-carriers, 749 E4-NCs
C	Mixed	1100	351 E4-carriers, 749 E4-NCs
D	Mixed-Condensed	352	176 E4-carriers, 176 E4-NCs
E	Mixed-Condensed	749	351 E4-carriers, 398 E4-NCs

**Table 6:** Statistical measurements of the model discrepancies.

Training Group	Test Group	BAD	CU (STD)	AD (STD)	ID
A	E4-carriers	7.1	2.8	2.3	**0.787**
B	E4-NCs	7.4	3.0	3.3	**0.738**
C	E4-carriers	8.3	3.2	2.7	**0.807**
C	E4-NCs	7.9	3.1	3.5	**0.733**
D	E4-carriers	7.1	2.9	2.3	**0.787**
D	E4-NCs	5.1	3.2	3.0	**0.581**
E	E4-carriers	7.8	3.1	2.5	**0.804**
E	E4-NCs	7.05	3.5	3.2	**0.687**

**Table 7:** Training group compositions for the gender comparison.

Training Group	Training Method	Training Size	Training Group Makeup
A	Gender-Specific	705	705 Females, 0 Males
B	Gender-Specific	395	0 Females, 395 Males
C	Mixed	1100	705 Females, 395 Males
D	Condensed-Mixed	706	353 Females, 353 Males
E	Condensed-Mixed	396	198 Females, 198 Males

**Table 8:** Performance statistics of random forest.

Training Group	Test Group	BAD	CU (STD)	AD (STD)	ID
A	Females	8.0	3.2	2.9	**0.789**
B	Males	7.7	2.9	3.0	**0.779**
C	Females	8.5	3.3	2.7	**0.816**
C	Males	8.1	2.9	3.2	**0.783**
D	Females	7.4	3.4	2.6	**0.766**
D	Males	8.0	3.2	3.3	**0.743**
E	Females	6.3	3.0	2.5	**0.746**
E	Males	7.1	2.8	3.1	**0.756**

## Data Availability

Data used in this article were collected from the National Alzheimer’s Coordinating Center (NACC) database (https://naccdata.org/). The code used for the models’ construction and analysis is available here: (CarterWo/MRI-data-analysis-for-AD-detection (github.com)).

## Supplementary Materials

The following supporting information can be downloaded at: https://www.mdpi.com/article/10.3390/bioengineering11090943/s1, Table S1: MRI feature list.
