# Supplementary material for: Machine Learning-Driven Prediction of Brain Age for Alzheimer’s Risk: APOE4 Genotype and Gender Effects
Source: Bioengineering (Basel). 2024 Sep 20;11(9):943. doi: 10.3390/bioengineering11090943 (PMC11429338; doi:10.3390/bioengineering11090943)
Supplement: Supplementary file 1 [file bioengineering-11-00943-s001.zip › bioengineering-3174681-supplementary.pdf]

Table S1. MRI feature list

Feature Description	P-Value between AD and CU values	CU Mean	CU STD	AD Mean	AD STD
Right entorhinal mean cortical thickness (mm)	<0.001*	3.76	0.58	2.795	0.864
Left entorhinal mean cortical thickness (mm)	<0.001*	3.535	0.616	2.732	0.799
Segmented total hippocampi volume (cc)	<0.001*	6.313	0.744	5.368	1
Segmented left hippocampus volume (cc)	<0.001*	3.124	0.383	2.636	0.517
Left isthmus cingulate mean cortical thickness (mm)	<0.001*	2.305	0.302	1.968	0.352
Segmented right hippocampus volume (cc)	<0.001*	3.19	0.395	2.732	0.525
Right superior temporal mean cortical thickness (mm)	<0.001*	2.228	0.295	1.903	0.364
Right isthmus cingulate mean cortical thickness (mm)	<0.001*	2.335	0.311	1.997	0.38
Right fusiform mean cortical thickness (mm)	<0.001*	2.564	0.476	2.127	0.422
Left superior temporal mean cortical thickness (mm)	<0.001*	2.12	0.252	1.851	0.334
Segmented total lateral ventricle volume (cc)	<0.001*	33.397	18.022	53.696	27.311
Segmented right lateral ventricle volume (cc)	<0.001*	15.944	8.638	25.735	13.425
Segmented total temporal lobe cortical gray matter volume (cc)	<0.001*	118.841	12.77	106.427	14.913
Right entorhinal gray matter volume (cc)	<0.001*	3.979	0.606	3.374	0.756
Segmented left lateral ventricle volume (cc)	<0.001*	17.452	9.681	27.956	14.729
Left inferior temporal gray matter volume (cc)	<0.001*	9.55	1.384	8.261	1.532
Segmented right temporal lobe cortical gray matter volume (cc)	<0.001*	58.001	6.39	51.898	7.6
Left inferior temporal mean cortical thickness (mm)	<0.001*	2.783	0.496	2.34	0.499
Segmented left temporal lobe cortical gray matter volume (cc)	<0.001*	60.549	6.572	54.246	7.911
Left fusiform mean cortical thickness (mm)	<0.001*	2.483	0.407	2.127	0.388
Right inferior temporal gray matter volume (cc)	<0.001*	11.062	1.581	9.655	1.646
Left entorhinal gray matter volume (cc)	<0.001*	4.125	0.61	3.577	0.74
Right parahippocampal mean cortical thickness (mm)	<0.001*	1.893	0.307	1.641	0.347
Right inferior temporal mean cortical thickness (mm)	<0.001*	2.821	0.51	2.423	0.524
Right middle temporal gray matter volume (cc)	<0.001*	12.482	1.662	11.136	1.898
Right precuneus mean cortical thickness (mm)	<0.001*	1.976	0.236	1.772	0.333
Left parahippocampal mean cortical thickness (mm)	<0.001*	1.807	0.297	1.58	0.321
Total cerebrum cerebrospinal ßuid volume (cc)	<0.001*	290.022	51.121	328.672	54.295
Left middle temporal gray matter volume (cc)	<0.001*	12.462	1.769	11.107	1.946
Right lingual mean cortical thickness (mm)	<0.001*	1.753	0.383	1.474	0.379
Right medial orbitofrontal mean cortical thickness (mm)	<0.001*	2.367	0.374	2.095	0.369
Right superior temporal gray matter volume (cc)	<0.001*	13.719	1.723	12.39	2.015
Left middle temporal mean cortical thickness (mm)	<0.001*	2.484	0.389	2.203	0.391
Left superior temporal gray matter volume (cc)	<0.001*	15.479	1.983	13.994	2.209
Right posterior cingulate mean cortical thickness (mm)	<0.001*	2.47	0.316	2.228	0.374
Right lateral orbitofrontal mean cortical thickness (mm)	<0.001*	2.491	0.376	2.225	0.367
Left lateral orbitofrontal mean cortical thickness (mm)	<0.001*	2.399	0.35	2.152	0.341
Left superior frontal mean cortical thickness (mm)	<0.001*	2.186	0.357	1.923	0.393
Left posterior cingulate mean cortical thickness (mm)	<0.001*	2.263	0.284	2.048	0.336

Right rostral anterior cingulate mean cortical thickness (mm)	<0.001*	3.047	0.472	2.703	0.516
Right superior frontal mean cortical thickness (mm)	<0.001*	2.296	0.344	2.055	0.355
Left rostral anterior cingulate mean cortical thickness (mm)	<0.001*	3.057	0.437	2.736	0.511
Left medial orbitofrontal mean cortical thickness (mm)	<0.001*	2.376	0.345	2.135	0.365
Segmented total third ventricle volume (cc)	<0.001*	1.367	0.539	1.738	0.581
Right parahippocampal gray matter volume (cc)	<0.001*	4.063	0.492	3.72	0.557
Total brain cerebrospinal ßuid volume (cc)	<0.001*	350.424	59.01	390.282	61.85
Left lingual mean cortical thickness (mm)	<0.001*	1.674	0.37	1.427	0.391
DEP2YRS_1.0	<0.001*	0.099	0.299	0.339	0.474
Right pars orbitalis mean cortical thickness (mm)	<0.001*	2.085	0.397	1.84	0.407
Left parahippocampal gray matter volume (cc)	<0.001*	3.925	0.468	3.62	0.56
Right insula mean cortical thickness (mm)	<0.001*	3.324	0.432	3.064	0.441
Right precuneus gray matter volume (cc)	<0.001*	9.293	1.343	8.489	1.487
Right paracentral mean cortical thickness (mm)	<0.001*	1.594	0.384	1.376	0.379
Right middle temporal mean cortical thickness (mm)	<0.001*	2.476	0.383	2.255	0.402
Right fusiform gray matter volume (cc)	<0.001*	7.918	1.269	7.201	1.284
Total cerebrum gray matter volume (cc)<br>Researchers Data Dictionary Ñ Imaging Data Page 22	<0.001*	491.392	53.903	460.513	56.98
Left precuneus mean cortical thickness (mm)	<0.001*	1.913	0.248	1.75	0.359
Left insula mean cortical thickness (mm)	<0.001*	3.302	0.434	3.057	0.458
Left caudal anterior cingulate mean cortical thickness (mm)	<0.001*	2.502	0.39	2.281	0.432
APOE4	<0.001*	0.319	0.466	0.576	0.495
Left transverse temporal mean cortical thickness (mm)	<0.001*	1.498	0.35	1.306	0.381
Segmented total parietal lobe cortical gray matter volume (cc)	<0.001*	95.444	11.769	89.162	12.36
Segmented right parietal lobe cortical gray matter volume (cc)	<0.001*	48.069	6.006	44.835	6.482
Left insula gray matter volume (cc)	<0.001*	6.031	0.74	5.636	0.827
Segmented left parietal lobe cortical gray matter volume (cc)	<0.001*	47.261	5.994	44.184	6.298
Left precuneus gray matter volume (cc)	<0.001*	9.61	1.446	8.861	1.582
Left fusiform gray matter volume (cc)	<0.001*	9.346	1.431	8.608	1.556
Right pericalcarine mean cortical thickness (mm)	<0.001*	1.189	0.361	1.015	0.344
Right inferior parietal gray matter volume (cc)	<0.001*	12.292	1.947	11.339	2.055
Right superior parietal gray matter volume (cc)	<0.001*	10.562	1.745	9.734	1.8
Right medial orbitofrontal gray matter volume (cc)	<0.001*	4.519	0.716	4.182	0.75
Total brain gray matter volume (cc)	<0.001*	589.029	60.955	560.353	63.716
Right lateral orbitofrontal gray matter volume (cc)	<0.001*	8.39	1.018	7.9	1.138
Left precentral mean cortical thickness (mm)	<0.001*	1.65	0.36	1.487	0.357
Right insula gray matter volume (cc)	<0.001*	6.236	0.735	5.882	0.858
Right supramarginal mean cortical thickness (mm)	<0.001*	1.84	0.278	1.709	0.322
Left superior frontal gray matter volume (cc)	<0.001*	22.43	3.868	20.702	4.014
Left caudal middle frontal mean cortical thickness (mm)	<0.001*	1.944	0.447	1.742	0.483
Right cuneus mean cortical thickness (mm)	<0.001*	1.393	0.28	1.263	0.327
Right pars opercularis mean cortical thickness (mm)	<0.001*	1.986	0.282	1.857	0.318

Right superior parietal mean cortical thickness (mm)	<0.001*	1.526	0.289	1.391	0.338
Right caudal anterior cingulate mean cortical thickness (mm)	<0.001*	2.736	0.419	2.549	0.442
Left isthmus cingulate gray matter volume (cc)	<0.001*	2.725	0.468	2.516	0.533
Left paracentral mean cortical thickness (mm)	<0.001*	1.555	0.375	1.395	0.391
Left Medial Orbitofrontal Gray Matter Volume (cc)	<0.001*	4.355	0.689	4.065	0.696
Left inferior parietal gray matter volume (cc)	<0.001*	11.21	1.946	10.4	1.965
Left superior parietal gray matter volume (cc)	<0.001*	10.422	1.666	9.732	1.667
Left lateral occipital gray matter volume (cc)	<0.001*	10.346	1.809	9.567	2.009
Segmented right frontal lobe cortical gray matter volume (cc)	<0.001*	85.649	10.538	81.163	11.619
Segmented total frontal lobe cortical gray matter volume (cc)	<0.001*	171.633	21.361	162.559	23.696
Segmented left frontal lobe cortical gray matter volume (cc)	<0.001*	85.242	10.745	80.641	12.19
Left supramarginal gray matter volume (cc)	<0.001*	10.785	1.718	10.09	1.694
Left lateral occipital mean cortical thickness (mm)	<0.001*	1.749	0.363	1.598	0.384
Segmented right occipital lobe cortical gray matter volume (cc)	<0.001*	30.311	4.591	28.395	4.967
Right superior frontal gray matter volume (cc)	<0.001*	24.73	4.037	23.096	4.123
Right precentral mean cortical thickness (mm)	<0.001*	1.643	0.336	1.514	0.318
Segmented total occipital lobe cortical gray matter volume (cc)	<0.001*	59.835	8.592	56.424	9.317
Right lateral occipital gray matter volume (cc)	<0.001*	10.271	1.818	9.561	2.023
Left lateral orbitofrontal gray matter volume (cc)	<0.001*	7.951	0.999	7.566	1.115
INCONTU_1.0	<0.001*	0.098	0.298	0.226	0.419
Left pars orbitalis mean cortical thickness (mm)	<0.001*	2.092	0.34	1.964	0.387
Left postcentral mean cortical thickness (mm)	<0.001*	1.43	0.265	1.335	0.263
Right caudal middle frontal mean cortical thickness (mm)	<0.001*	1.955	0.387	1.813	0.433
Right inferior parietal mean cortical thickness (mm)	<0.001*	1.945	0.334	1.826	0.349
SEX	<0.001*	1.641	0.48	1.47	0.5
Right lateral occipital mean cortical thickness (mm)	<0.001*	1.781	0.369	1.649	0.394
Right paracentral gray matter volume (cc)	<0.001*	4.059	0.871	3.755	0.881
Left pericalcarine mean cortical thickness (mm)	<0.001*	1.094	0.322	0.982	0.338
Left pars opercularis mean cortical thickness (mm)	<0.001*	1.896	0.262	1.797	0.338
NACCBMI	<0.001*	26.856	6.464	24.462	7.994
Segmented left occipital lobe cortical gray matter volume (cc)	<0.001*	29.414	4.329	27.887	4.79
Right isthmus cingulate gray matter volume (cc)	<0.001*	2.721	0.499	2.547	0.534
Left transverse temporal gray matter volume (cc)	<0.001*	0.89	0.244	0.812	0.222
Left superior parietal mean cortical thickness (mm)	<0.001*	1.623	0.273	1.526	0.333
Total cerebrum brain volume (cc)	<0.001*	890.572	98.464	859.041	95.021
Right transverse temporal mean cortical thickness (mm)	<0.001*	1.467	0.373	1.342	0.416
Total brain white matter hyperintensity volume (cc)	<0.001*	6.701	11.265	10.899	15.818
Left precentral gray matter volume (cc)	<0.001*	12.273	2.234	11.564	2.357
Right postcentral mean cortical thickness (mm)	<0.001*	1.433	0.246	1.358	0.231
Left caudal middle frontal gray matter volume (cc)	<0.001*	4.624	1.061	4.308	1.005
Total brain volume (cc)	<0.001*	1020.652	112.081	987.283	107.671

Right supramarginal gray matter volume (cc)	<0.001*	8.647	1.545	8.179	1.618
Left supramarginal mean cortical thickness (mm)	<0.001*	2.022	0.275	1.94	0.309
Right pericalcarine gray matter volume (cc)	<0.001*	2.615	0.641	2.434	0.643
Left pars opercularis gray matter volume (cc)	<0.001*	4.327	0.845	4.084	0.883
Right caudal middle frontal gray matter volume (cc)	<0.001*	6.269	1.285	5.907	1.312
Left cuneus mean cortical thickness (mm)	<0.001*	1.342	0.28	1.259	0.334
Right cuneus gray matter volume (cc)	<0.001*	4.115	0.83	3.884	0.918
Right posterior cingulate gray matter volume (cc)	<0.001*	3.895	0.651	3.722	0.651
Right pars triangularis mean cortical thickness (mm)	<0.001*	1.89	0.35	1.802	0.343
Left paracentral gray matter volume (cc)	<0.001*	4.079	0.884	3.857	0.873
Left inferior parietal mean cortical thickness (mm)	<0.001*	2.071	0.33	1.986	0.353
Left rostral middle frontal mean cortical thickness (mm)	<0.001*	2.13	0.411	2.027	0.416
Right pars orbitalis gray matter volume (cc)	<0.001*	1.827	0.419	1.725	0.4
Right precentral gray matter volume (cc)	<0.001*	11.327	1.938	10.849	2.08
Total white matter volume (cc)	<0.001*	386.556	151.131	348.452	184.445
Total intracranial volume (cc)	<0.001*	1212.466	460.489	1097.365	576.153
Left rostral middle frontal gray matter volume (cc)	<0.001*	10.301	1.689	9.914	1.797
Right rostral middle frontal mean cortical thickness (mm)	<0.001*	2.148	0.409	2.057	0.412
Left postcentral gray matter volume (cc)	<0.001*	8.986	1.634	8.629	1.589
Right transverse temporal gray matter volume (cc)	<0.001*	0.857	0.247	0.804	0.24
Left posterior cingulate gray matter volume (cc)	<0.001*	4.073	0.632	3.936	0.706
EDUC	<0.001*	15.495	3.594	14.736	3.81
Left pars triangularis mean cortical thickness (mm)	<0.001*	1.879	0.306	1.816	0.335
Right pars opercularis gray matter volume (cc)	<0.001*	4.372	0.839	4.207	0.834
Right rostral middle frontal gray matter volume (cc)	<0.001*	10.678	1.763	10.328	1.813
Left lingual gray matter volume (cc)	<0.001*	6.533	1.121	6.311	1.189
Right postcentral gray matter volume (cc)	<0.001*	8.769	1.598	8.468	1.53
Right caudal anterior cingulate gray matter volume (cc)	<0.001*	1.964	0.537	2.059	0.549
Right lingual gray matter volume (cc)	0.002	7.256	1.333	7.041	1.385
Right pars triangularis gray matter volume (cc)	0.002	3.961	0.877	3.826	0.845
Right rostral anterior cingulate gray matter volume (cc)	0.004	2.482	0.564	2.401	0.533
VISWCORR_1.0	0.008	0.785	0.411	0.728	0.446
SMOKYRS	0.011	7.022	13.009	8.739	13.871
NACCDBMD_1.0	0.03	0.128	0.334	0.093	0.291
Left pericalcarine gray matter volume (cc)	0.036	2.534	0.671	2.461	0.707
Left cuneus gray matter volume (cc)	0.037	4.295	0.911	4.195	1.014
BPDIAS	0.048	72.03	15.762	70.369	17.937
HRATE	0.058	65.483	16.3	63.867	17.593
Total cerebrum white matter volume (cc)	0.076	392.53	55.481	387.667	51.027
Left pars orbitalis gray matter volume (cc)	0.077	2.008	0.44	1.968	0.457
Left pars triangularis gray matter volume (cc)	0.086	3.942	0.844	3.866	0.922
B12DEF_1.0	0.09	0.032	0.176	0.048	0.214
NACCLIPL_1.0	0.101	0.5	0.5	0.458	0.499
Total brain white matter volume (cc)	0.118	431.623	61.156	426.93	55.608

Left rostral anterior cingulate gray matter volume (cc)	0.258	3.621	0.723	3.578	0.796
Total cerebrum cranial volume (cc)	0.277	1180.594	126.766	1187.713	133.021
CBTIA_1.0	0.301	0.018	0.134	0.012	0.107
BPSYS	0.358	130.277	28.458	128.862	33.523
B12DEF_2.0	0.364	0.017	0.13	0.012	0.107
DIABETES_1.0	0.364	0.125	0.33	0.11	0.313
SOMATIC_1.0	0.377	0.019	0.137	0.013	0.115
NACCBETA_1.0	0.604	0.185	0.388	0.174	0.38
Left caudal anterior cingulate gray matter volume (cc)	0.778	3.29	0.761	3.302	0.809
NACCAGE	0.935	76.078	8.341	76.113	8.491
